# Prone position surgery for a subcarinal bronchogenic cyst

**DOI:** 10.1186/s40792-018-0557-6

**Published:** 2018-12-29

**Authors:** Toru Nakamura, Ryo Fujikawa, Yoshiro Otsuki, Kazuhito Funai

**Affiliations:** 1 Department of General Thoracic Surgery, Seirei Hamamatsu General Hospital, 2-12-12 Sumiyoshi, Hamamatsu, Shizuoka Japan; 2 Department of Pathology, Seirei Hamamatsu General Hospital, 2-12-12 Sumiyoshi, Hamamatsu, Shizuoka Japan; 3 First Department of Surgery, Hamamatsu University school of Medicine, 1-20-1 Handa-yama, Hamamatsu, Shizuoka Japan

**Keywords:** Prone position, Thoracoscopic surgery, Bronchogenic cyst

## Abstract

**Background:**

Prone position surgery has become widespread for esophageal cancer instead of the traditional lateral decubitus approach. Carbon dioxide insufflation and the gravity effect provide a better operative field without parenchymal retraction. We herein report a case of a subcarinal bronchogenic cyst, which was successfully removed by the prone position surgery.

**Case presentation:**

A 65-year-old man presented with a subcarinal mass and was planned to undergo a surgical resection in the prone position. Although he required bilateral ventilation due to hypoxemia, the excellent operative field was maintained and we completed the thoracoscopic surgery without any additional parenchymal retractions.

**Conclusions:**

Thoracoscopic surgery in the prone position is a feasible option for subcarinal tumors with an excellent operative view and would facilitate a solo surgery without the need for a skilled assistant.

## Background

Bronchogenic cysts are rare congenital neoplasms arising from abnormal budding of the bronchial tree and often develop in the mediastinum. Further, video-assisted thoracoscopic surgery (VATS) in the lateral decubitus position has been the standard surgical approach for mediastinal tumors to date [1–3]. In addition, recent reports have suggested the utility of the prone position approach with carbon dioxide (CO_2_) insufflation for esophageal cancer and other mediastinal tumors [4–6]. We herein report a case with a subcarinal bronchogenic cyst that successfully underwent a thoracoscopic resection in the prone position.

## Case presentation

**Fig. 1:**
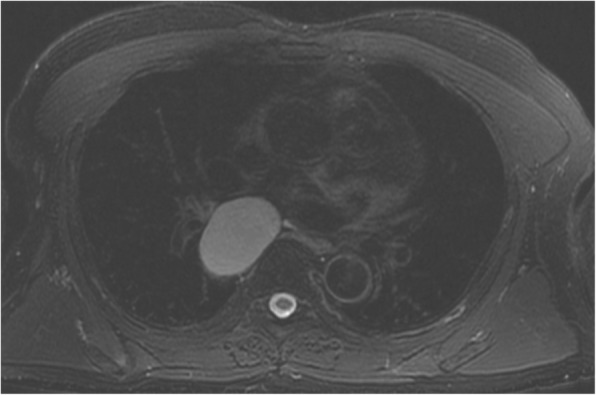
Axial T2 weighted image showing a cyst in the subcarinal area

A 65-year-old man presented with a subcarinal mass incidentally found during the preoperative work up for bladder cancer. Magnetic resonance imaging revealed a circumscribed mass suggesting a bronchogenic cyst (Fig. 1). After completing a trans-urethral resection of bladder cancer, he was referred to our department and planned to undergo a surgical resection of the tumor.

**Fig. 2:**
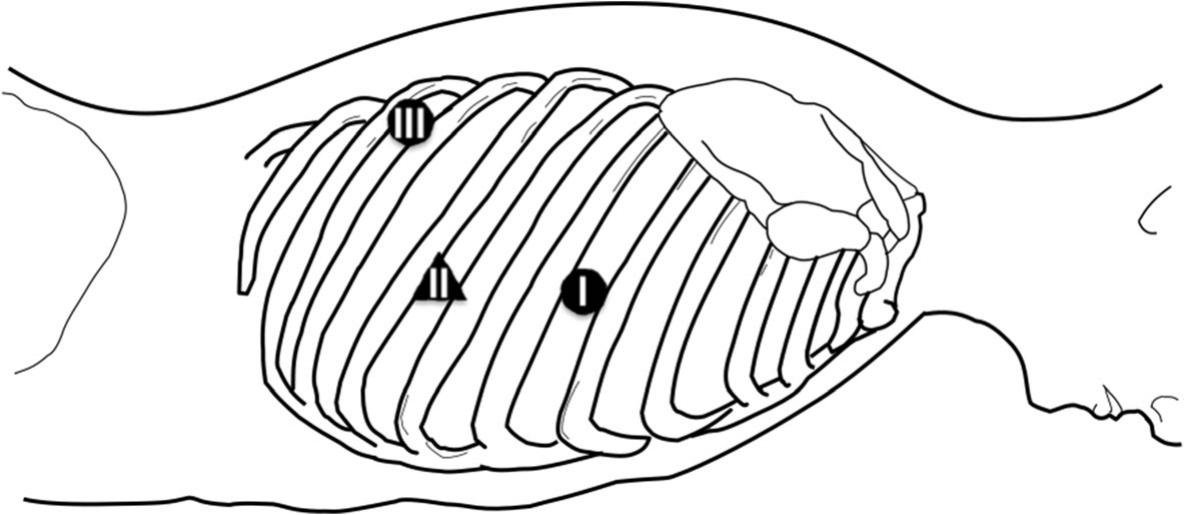
Patient position and port insertion site

The patient was placed in the prone position and his right arm was raised cranially. Under one-lung ventilation, the initial 5-mm port was inserted at the fifth intercostal space (ICS) on the right middle axillary line (port 1, Fig. 2). CO_2_ was insufflated through this port at a pressure of 8 mmHg. Under a thoracoscopic view, the second 5-mm port and third 12-mm port were inserted at the ninth ICS on the scapular line (port 2) and seventh ICS on the middle axillary line (port 3), respectively. With the thoracoscope inserted through port 2, the surgeon held a grasper and electrocautery via port 3 and port 1.

**Fig. 3:**
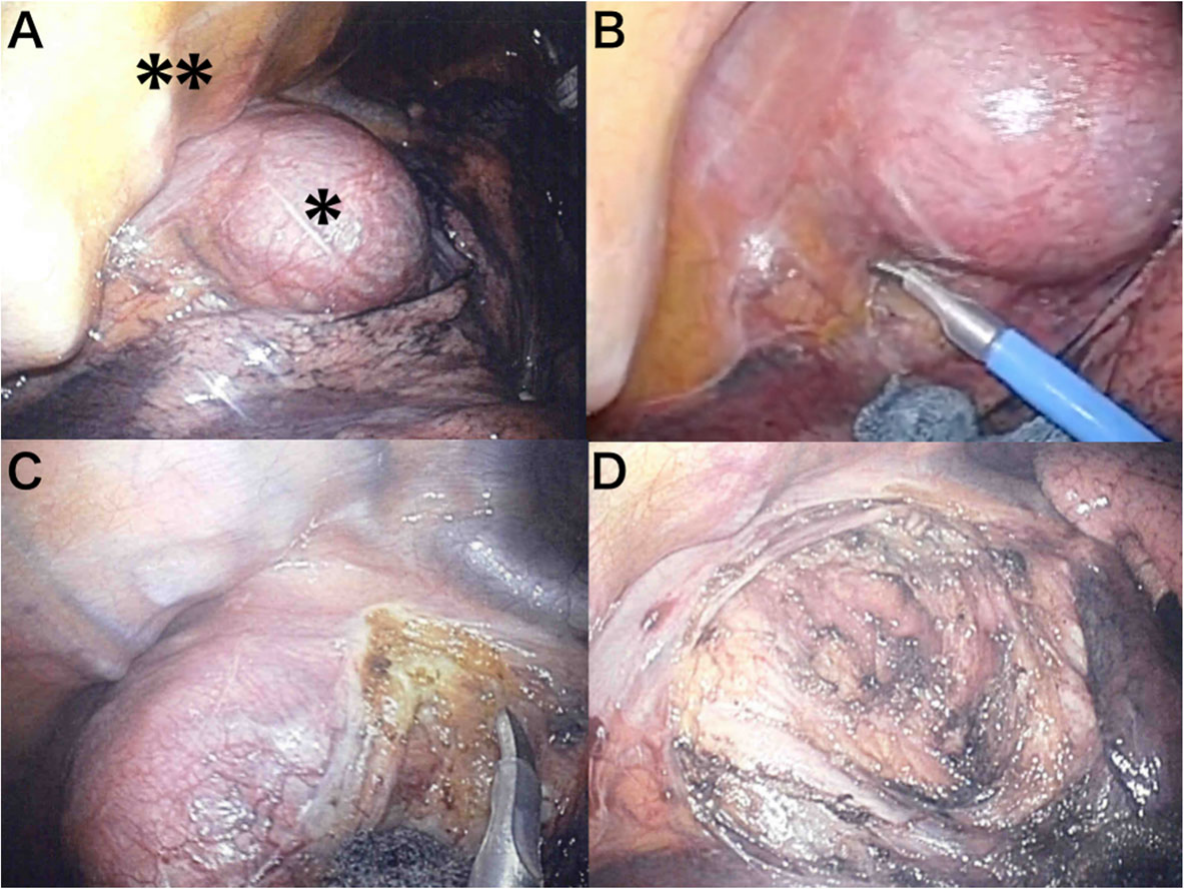
Operative view in the prone position. **a** Carbon dioxide insufflation facilitated the exposure of the tumor (*) anterior to the thoracic vertebra (**). **b** The mediastinal pleura was incised inferiorly to mobilize the tumor from the pericardium. **c** By dissecting the pleura toward the cranial direction, the tumor was removed from the bronchus and esophagus. An excellent operative field was maintained even with bilateral ventilation. **d** The tumor was completely removed

In addition to CO_2_ insufflation, a gravity effect also facilitated the exposure of the posterior mediastinum and subcarinal mass wide enough without any retraction (Fig. 3a). The mediastinal pleura was incised inferiorly to mobilize the tumor from the pericardium and bronchus (Fig. 3b, c).

However, 10 min after beginning the surgery, he required bilateral ventilation because of hypoxemia when his O_2_ saturation dropped to 81% probably caused by diaphragmatic compression (his body mass index was 25.3). Nevertheless, the operative field was maintained excellently even with bilateral ventilation and we completed the total resection of the tumor successfully (Fig. 3d). The operative time and estimated blood loss were 126 min and 1 g, respectively. A histological examination revealed that the cyst wall lined by pseudostratified ciliated epithelium contained smooth muscle, consistent with a diagnosis of a bronchogenic cyst. The postoperative course was uneventful, and he was discharged 3 days after surgery.

## Conclusions

Bronchogenic cysts often develop in the mediastinum and require a surgical resection even in asymptomatic patients to prevent complications such as infections, hemorrhage, raptures, and malignant formations [7–10] . Although the lateral decubitus VATS approach has been the standard approach for mediastinal tumors over the decades, we performed the surgery in the prone position in our case. Compared with the traditional lateral decubitus VATS approach, the prone position surgery provides easier access to the posterior mediastinum mainly by the gravity effect. Combined with positive pressure by CO_2_ insufflation, the lung and heart fall downward and the blood also drips outside the operative field. These factors provided an excellent surgical view without any additional retraction and enabled a solo surgery in our case.

Although the usefulness of a bilateral approach in the decubitus position for subcarinal bronchogenic cysts has been reported, it was deemed to be troublesome because a positioning change of the patient was required during the surgery [11]. Further, the prone position has been reported to facilitate a better lymph node dissection in the subcarinal area than a lateral decubitus position during esophagectomy [12]; however, a unilateral approach in the prone position would be a feasible option for surgery for subcarinal tumors. In addition, the prone position surgery also allowed a left-sided and bilateral approach without any morbidity [13, 14]. These results suggested that the prone position approach would be more beneficial both for the curability and utility than the lateral decubitus position for the posterior mediastinum surgery. We consider that this surgical approach would be applicable for other types of posterior mediastinal tumors, such as schwannoma located in the superior mediastinum.

Thoracoscopic surgery in the prone position is a feasible option for subcarinal tumors. CO_2_ insufflation and the gravity effect provide an excellent operative view without any additional parenchymal retraction and might facilitate a solo surgery even with bilateral ventilation.

## Abbreviations

CO_2_: Carbon dioxide
ICS: Intercostal space
VATS: Video-assisted thoracoscopic surgery
